# Effects of Melatonin on Dairy Herd Improvement (DHI) of Holstein Cow with High SCS

**DOI:** 10.3390/molecules26040834

**Published:** 2021-02-05

**Authors:** Hao Wu, Songyang Yao, Tiankun Wang, Jun Wang, Kang Ren, Hai Yang, Wenkui Ma, Pengyun Ji, Yongqiang Lu, Hui Ma, Changwang He, Wenjuan Wei, Lu Zhang, Guoshi Liu

**Affiliations:** 1 National Engineering Laboratory for Animal Breeding, Key Laboratory of Animal Genetics and Breeding of the Ministry of Agricultural, Beijing Key Laboratory for Animal Genetic Improvement, College of Animal Science and Technology, China Agricultural University, Beijing 100193, China; 18800160525@163.com (H.W.); songyangyao@cau.edu.cn (S.Y.); Yanghai19940315@163.com (H.Y.); Ma17610890927@163.com (W.M.); jipengyun1989@126.com (P.J.); luzhang2018@cau.edu.cn (L.Z.); 2 Beijing Chang Ping District Animal Disease Prevention and Control Center, Beijing 102200, China; cpfys8016@163.com; 3 Beijing Animal Husbandry and Veterinary General Station, Beijing 100012, China; wj552510@163.com (J.W.); renkang024@163.com (K.R.); luyongqiang@163.com (Y.L.); 4 Beijing Shou Nong Food Group Co. Ltd., Beijing 100029, China; mary_2291@163.com (H.M.); hechangwang9160@sina.com (C.H.); xiayiwwj@sina.com (W.W.)

**Keywords:** melatonin, milk, cows, somatic cell score, season, age, lactation period

## Abstract

Mastitis is a common disease in cows breeding. The milk quality will be significantly reduced with increased milk somatic cells, which often occurs in cows with mastitis. In this study, the influence of seasonal changes, age and lactation stages in the Dairy Herd Improvement (DHI) of cows was investigated. Then, the Dairy Herd Improvement (DHI) of cows with high somatic cell score (SCS) after melatonin treatment was systemically investigated. The results showed that melatonin significantly suppressed the milk somatic cell score under all of the tested conditions. The melatonin treatment also improved the milk nutritional value by reducing its fat but increasing its lactose and protein contents. The application of melatonin significantly improved the DHI. The beneficial effects of melatonin on DHI are likely attributed to the antioxidant and anti-inflammatory activities of melatonin.

## 1. Introduction

DHI (Dairy Herd Improvement) is a standard measurement used to evaluate the milk productive performance of dairy cows [1]. DHI measurements include the parameters of the milk somatic cell number (SCC), milk yield, protein, fat, lactose, dry matter and urea nitrogen of the milk [2]. DHI establishment is an important approach to improve the dairy herd performance and to breed high-yielding cow herds [3]. Among the parameters measured in the DHI, the SCC is a most important indicator to reflect the milk quality and the status of breast health of the cows [4]. Many factors influence the milk SCC. These include environmental stresses, number of fetuses, air humidity, feeding management, nutrition level, etc. [5,6]. Among the environmental stresses, the seasonal alterations have a significant impact on the SCC [7]. For example, high temperatures (hot stress) in the summer often causes reduced milk production and elevated SCC [8]. Cows in different ages exhibit variable DHI indexes [9]. This partially relates to the fact that cows are sexually matured at the first mating time, but they still do not reach fully physical maturity. Thus, with the continuous body development, the somatic cell score exhibits regular changes with the gestational time [10]. In addition, cows in the different lactation periods, including the early, peak, middle and late lactation periods, have different milk production and physiological characteristics, which also result in variable DHI values. For example, the SCC in the early stage of lactation is higher than that in the peak and middle stages of lactation [11].

SCC, lactose, fat, protein and urea nitrogen indexed in the DHI determine the milk quality [12]. The higher the SCC, the lower the milk quality is [13]. When the milk SCC exceeds 200,000/mL, the cow would be diagnosed as having mastitis. An extremely high SCC always indicates a serious breast infection of cows [14]. Lactose is a unique ingredient in milk and is present in many infant formulas and dairy products [15]. Milk fat is a complex mixture of different types of fat, which is the main source of energy of the milk [16]. The milk proteins are mainly casein, with small amounts of albumin and globulin [17], which is an important protein source of the human diet. Urea nitrogen reflects the efficiency of nitrogen and protein metabolism in rumen. The urea nitrogen content provides accurate information as to how much milk should be consumed based on the daily protein diet requirements [18]. Changes in any of these factors mentioned above will affect the quality of the raw milk and the quality of its processed products [19]. Milk with a balanced composition ratio of these factors is considered to have suitable nutritional value [20].

To produce high-quality milk with low SCC is a priority agenda for cow breeders as well as researchers. In a previous study, Yang M. et al. found that a melatonin application reduced milk SCC [21]. This study is to investigate whether a melatonin application can increase the milk nutritional value. This aspect has not been reported until now.

Melatonin (MT) was originally classified as an endocrine hormone mainly produced by the pineal gland in vertebrates. Currently, it is found that many cells, organs and tissues have the capacity to synthesize melatonin. These include the gastrointestinal tract [22], retina [23], bone marrow [24], skin [25], oocytes [26], etc. The main sites of melatonin synthesis are the mitochondria [27], and thus, actually, all cells appear to have the ability to synthesis melatonin. Melatonin production exhibits an apparent circadian rhythm, with the peak at dark and the baseline level during the day [28]. Melatonin administration during a dry period is galactopoietic for the subsequent lactation in goats [29]. Few studies have reported the effects of melatonin supplement on milk quality in cows evaluated by the DHI, which is considered as the best index to reflect milk quality in the field. Thus, in the current study, we adopted the DHI to systemically evaluate melatonin’s effects on milk quality. The results are reported in the following section.

## 2. Results

### 2.1. Influence of Seasonal Changes on the Milk DHI Index

The DHI data of Holstein cows without melatonin treatment in different seasons showed that the highest somatic cell score (SCS) occurred in the summer, which was significantly higher (*p* < 0.05) than that in the winter (Table 1). The milk yield in the spring was significantly higher (*p* < 0.05) than that in the summer and autumn (Table 1). The protein content in the summer was the lowest (*p* < 0.05) compared to the spring, autumn and winter (Table 1). The lactose content in the autumn was significantly higher (*p* < 0.05) than that in the spring (Table 1). The dry matter content in the summer was significantly lower than that in the spring and autumn (Table 1). No significant differences were observed in the fat and urea nitrogen contents in different seasons (Table 1).

### 2.2. Effects of Melatonin on Milk DHI Index in Cows with High SCS during Different Seasons

Four consecutive days of melatonin injection significantly increased the levels of melatonin in the blood (Table 2). The profound effect of melatonin on milk SCS was observed in all seasons—that is, melatonin significantly reduced the SCS in different seasons, with the maximum effect in the summer (Table 3). The melatonin application significantlyincreased the protein content only in the autumn (*p* < 0.05) (Table 3). However, the melatonin application consistently increased the lactose content, with significant effects in the spring, summer and winter (*p* < 0.05) (Table 3). Interestingly, the melatonin treatment significantly reduced the milk fat content only in the spring (*p* < 0.05) (Table 3). The melatonin treatment had no significant effect on the milk production, dry matter and urea nitrogen in different seasons (Table 3).

### 2.3. Influence of Cow’s Age on Milk DHI Index

The DHI data of Holstein cows without the melatonin treatment of different ages showed that age is a reliable factor to impact the SCS. The SCS was gradually increased with age. The oldest cows (seven years old) had the greatest SCS values, and the youngest cows (two years old) exhibited the lowest among other age groups (*p* < 0.05) (Table 4). The highest milk production was observed in the three-year-old cows. The seven-year-old cows had the lowest milk yields among the other age groups (*p* < 0.05) (Table 4). The milk protein content was relatively high in cows four years old compared to the other age groups (Table 4). The milk lactose content decreased with age (Table 4). In contrast, the milk fat content increased with the age, but it rapidly declined in cows of seven years old (Table 4). No significant differences were identified in the dry matter and urea nitrogen contents among the different age groups (*p* > 0.05) (Table 4).

### 2.4. Effect of Melatonin on Milk DHI Index in Cows with High SCS of Different Ages

The application of melatonin significantly reduced the SCS in all age groups, except in the six-year-old group, in which this decrease did not reach a significant difference (Table 5). The results also showed that the melatonin treatment significantly increased the milk lactose content in cows at the three-year-old and four-year-old groups compared to rest of the age groups (Table 5). Milk fat was significantly reduced by the melatonin treatment only in cows of the three-year-old group compared to the other groups (Table 5). No significant differences were detected in the daily milk production, protein, dry matter content and urea nitrogen content among the different age groups after the melatonin treatment (Table 5).

### 2.5. Influence of Lactation Stages on the Milk DHI Index

The DHI data of Holstein cows without the melatonin treatment in different lactation stages showed that the lactation stages had a profound influence on the milk SCS. The SCS was significantly increased as the lactation stage advanced, with the highest SCS at the end of the lactation stage (Table 6). The milk yield in the middle lactation stage was significantly higher than that in the other stages (Table 6). The fat content in the early lactation stage was significantly higher than that in the end lactation stage (Table 6). No significant differences were detected in the milk protein, lactose, urea nitrogen content and dry matter in the different lactation stages (Table 6).

### 2.6. Effects of Melatonin on the DHI Index in Cows with High SCS of Different Lactation Stages

The melatonin treatment reduced the milk SCS in the cows at the early lactation stage, but this decrease did not reach a significant difference. However, the melatonin treatment significantly highly suppressed the SCS in the middle and end lactation stages compared to the controls (Table 7). The melatonin treatment significantly elevated the milk lactose content at the early and end lactation stages compared to the controls (Table 7). No significant differences were detected in the milk yield, protein, fat, dry matter and urea nitrogen during the different lactation stages after the melatonin treatment (Table 7).

### 2.7. A Comprehensive Analysis of the Effect of Melatonin on the DHI in Milk

**Figure 1:**
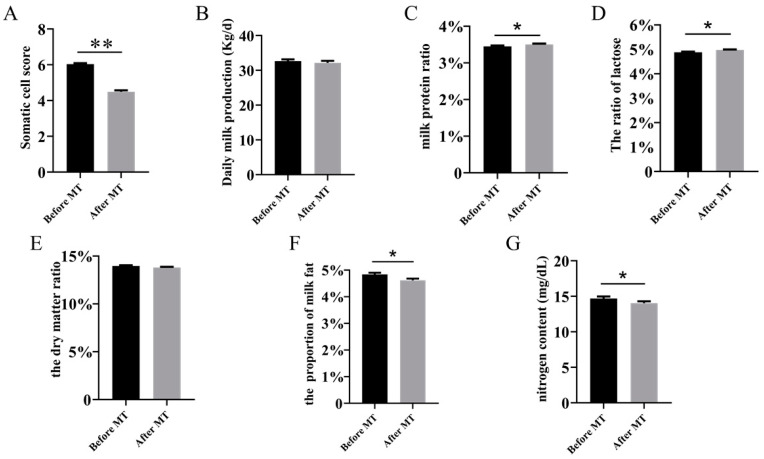
Influence of the melatonin application on the Dairy Herd Improvement (DHI). (**A**) Somatic cell score, (**B**) milk yield, (**C**) protein content, (**D**) lactose content, (**E**) fat content, (**F**) dry matter content and (**G**) urea nitrogen content. All data are represented by the mean ± standard error, * *p* < 0.05, ** *p* < 0.01, *n* = 328. MT: melatonin.

The DHI data after the melatonin treatment in different seasons were summarized and then analyzed comprehensively. The data showed that the subcutaneous injection of melatonin reduced the milk SCS (Figure 1A), fat content (Figure 1F), urea nitrogen content (Figure 1G) and increased milk protein content (Figure 1C) and lactose content (Figure 1D), respectively, compared to the untreated controls. No significant differences were detected in the daily milk production and milk dry matter after the melatonin application.

## 3. Discussion

How to evaluate the quality of fresh milk is a challenge for the dairy industry, since it is still debatable for the justification of the levels of nutrition and residues of antibiotics in milk [30]. Nevertheless, the DHI index was adopted by the entire dairy industry to evaluate the quality of milk [31]. The DHI index reflects the dairy production performance and fresh milk quality in individual cows. In the current study, we systemically investigated the influences of seasonal changes, age and lactation stages on the DHI in the cows with potential mastitis. Most importantly, in the paralleled studies, the effects of melatonin on the DHI were also studied under all of these environmental and physiological conditions. As we reported in the results, all of these alterations differentially impacted the DHI. For example, reduced milk protein, fat and dry matter were found in the summer (Figure 1). The most significant alteration caused by seasonal changes, age and lactation stage was the SCS. Summer, advanced ages and the end stage of lactation all significantly enhanced milk SCS. These results were consistent with previous reports. Green et al. reported the highest SCS being found in the summer among all the seasons [8]. Actually, animals exposed to a long photoperiod will drop their melatonin production in the summer [32]. This may be an additional reason to have a higher milk SCS in the summer. The increased SCS was also observed followed the increased lactation time due to the poor milking practices and breast management [33].

Milk SCS is an important index of breast health and milk quality. The higher the SCS, the lower the milk quality is [34]. Once the SCS was more than 3 × 10^5^ cells/mL, it indicated clinical mastitis of the cows and, also, increased the elimination rate of cows suffering from mastitis [35]. The use of antibiotics is the conventional treatment for mastitis, and it effectively reduces the SCS [36]. However, frequently, the use of antibiotics leads to drug resistance of the animals, and the residues of antibiotics in the milk are a safety concern for human health [37]. The identification of remedies that can replace antibiotics to target the mastitis is an urgent task for researchers. We realized that the major etiologies of mastitis are associated with oxidative stress and a proinflammatory reaction after infection [38]. Thus, in the current study, melatonin was selected for this purpose. Melatonin is a potent free radical scavenger and antioxidant [39]. It detoxifies a spectrum of reactive oxygen species (ROS) and reactive nitrogen species (RNS). It is also an anti-inflammatory molecule, and its anti-inflammatory activities have been well-documented [40]. The most notable change observed in this study was the suppressive effect of the melatonin application on the milk SCS. No matter in what conditions, including in the seasonal alterations, increased age and advanced lactation stage, melatonin uniformly and significantly reduced the milk SCS compared to the untreated controls. A systematic analysis of the melatonin application to the cows yielded the remarkable result that melatonin significantly suppressed the milk SCS (Figure 1). The SCS suppressive effect of melatonin was consistent with the previous report, which showed that the subcutaneous injection of melatonin lowered the SCS and cortisol level in cows with subclinical mastitis [21].

It is known that the majority of milk somatic cells are derived from neutrophils [41]. Many studies have reported that melatonin has the capacity to reduce neutrophil infiltration into the infected tissue and then subside the inflammatory reaction [42]. This may also be applied to mastitis and reducing the milk SCS with a melatonin treatment. In addition, oxidative stress is an important promoter of mastitis, and melatonin has been reported to lower the oxidative stress in the mastitis of cows [43]. Thus, the milk SCS lowering effect is mainly attributed to the antioxidative and anti-inflammatory activities of melatonin [44]. In this study, we also observed that the application of melatonin modified some nutritional compositions of the milk. It reduced the milk fat but increased the milk lactose and protein contents. Milk fat is mainly composed of triglycerides with nutritional value [45], and the modification of its content is also the main target in dairy breeding [46]. Reports as to the effects of melatonin on milk fat are not consistent. Some have reported that melatonin had no effect on the milk composition [47], and others showed that melatonin indeed reduced the amount of fat in mid-stage lactating milk [48]. In this study, we observed that melatonin decreased the milk fat content during different seasons, ages and lactation periods. A molecular mechanism study identified that melatonin inhibits the mTOR signaling pathway through MT1 receptors in bovine mammary epithelial cells, and via this pathway, melatonin suppresses the synthesis of milk fat [49]. This observation provides a new basis for applying melatonin to regulate milk fat metabolism.

Lipids, sugar and proteins can be transformed into each other in the body [50]. The decreased milk fat can be used to synthesize milk lactose and proteins [51] to improve the nutritional value of milk, as observed in this study. In conclusion, the influence of seasonal changes, age and lactation stages on the DHI of dairy cows was systemically studied. All of these factors impact the values of the DHI. An application of melatonin significantly improves the DHI under any condition mentioned above. The most profound effect of melatonin is to suppress the SCS. The systematic analysis of the application of melatonin to cows reduced the milk SCS by 63.54%. Such a great effect of melatonin on the milk SCS has not been reported previously. In addition, the application of melatonin improved the milk quality by reducing the fat and increasing the protein and lactose. The beneficial effects of melatonin are likely attributed to its antioxidative and anti-inflammatory activity. It seems that melatonin may be a suitable candidate to replace antibiotics to treat mastitis in dairy cows. For this purpose, further investigation is warranted.

## 4. Materials and Methods

### 4.1. Chemicals

Melatonin was purchased from Sigma Company (St. Louis, MO, USA), and anhydrous ethanol was purchased from Sinopharm Group Chemical Reagent Co., Ltd. (Shanghai, China). Melatonin was dissolved in ethanol and diluted with normal saline while in a dark room. The volume ratio of anhydrous ethanol to normal saline was 2:3, and the solvent was prepared with 4.64-mg/mL melatonin.

### 4.2. Animals

Holstein cows with normal reproductive cycles, disease-free and similar body conditions were selected for the study. The cows were fed with a Total Mixed Rations (TMR) diet (NRC2001) 3 times at 06:00, 12:00 and 18:00, respectively, and after each feeding, the cows were allowed to enter the outside yard for drink and free activities.

### 4.3. Study Design

Based on the monthly reports of the DHI measurements, cows with milk SCC 0.3–1 million/mL were selected for the studies. In the current study, the effects of seasonal changes were also considered as to the melatonin treatment on milk quality. Melatonin was injected subcutaneously at 10 a.m. with a dose of 9.3 mg/cow/day for 4 consecutive days. Before and 15 days after the melatonin injection, the fresh milk was collected for DHI analyses. The general information of the cows in the study is listed in Table 8 and Table 9.

### 4.4. DHI Measure

Forty milliliters of fresh milk were collected and mixed with 2 to 3 drops of saturated potassium dichromate solution and stored at 2–7 °C for further analysis. DHI determination was performed by the National Milk Product Standard Sanction Laboratory located at the Beijing Animal Husbandry Station by the use of a DHI measuring instrument (MilkoscanFT1, Hilleroed, Denmark).

### 4.5. Melatonin Assay

Three milliliters of serum from 20 cows were collected to detect the melatonin concentration before each melatonin injection. Melatonin detection was carried out in the central laboratory of the Beijing Institute of Animal Science, Chinese Academy of Agricultural Sciences by the use of the DHI measuring instrument (Agilent1290-G6470, Santa Clara, CA, USA).

### 4.6. Statistical Analysis

The somatic cell number (SCC) was converted into a somatic cell score (SCS) using the formula: SCS = log_2_(SCC/100) + 3 [52]. All data were presented as the mean ± SEM. One-way analysis of variance was used for the comparison of multiple means among the groups and a paired *t*-test for the comparison of a single mean between the melatonin treatment groups (SPSS software, version 25.0 (IBM SPSS Statistics, Armonk, NY, USA)). *p* < 0.05 was considered a significant difference.

## 5. Conclusions

In conclusion, our results indicated that melatonin can significantly reduce the SCS and improve the DHI in the milk of Holstein cows with high SCS. The melatonin treatment also improved the milk nutritional value by reducing its fat but increasing its lactose and protein contents. Considering the safety of melatonin with none or low side effects to animal or human health, the development of melatonin products and their application in dairy farming can improve the industrial benefits.

## Figures and Tables

**Table 1:** The influences of seasonal changes on the Dairy Herd Improvement (DHI) index in cows.

Items	Spring	Summer	Autumn	Winter
somatic cell score	4.5 ± 0.04 ^ab^	5.1 ± 0.46 ^a^	4.3 ± 0.20 ^ab^	3.8 ± 0.28 ^b^
milk yield (kg/d)	34.9 ± 0.14 ^a^	33.6 ± 0.19 ^b^	33.6 ± 0.33 ^b^	34.2 ± 0.22 ^ab^
Protein (%)	3.5 ± 0.02 ^a^	3.3 ± 0.03 ^b^	3.5 ± 0.02 ^a^	3.5 ± 0.05 ^a^
Lactose (%)	5.0 ± 0.02 ^b^	5.1 ± 0.02 ^a^	5.1 ± 0.01 ^a^	5.1 ± 0.07 ^a^
fat (%)	4.5 ± 0.17	4.4 ± 0.35	4.5 ± 0.01	4.4 ± 0.32
dry matter (%)	13.8 ± 0.13 ^a^	13.3 ± 0.17 ^b^	14.0 ± 0.02 ^a^	13.7 ± 0.23 ^ab^
urea nitrogen (mg/dL)	14.6 ± 1.98	14.3 ± 2.26	13.5 ± 1.11	16.6 ± 2.79

All data are represented by the mean ± standard error. *n* = 728 in the spring, *n* = 702 in the summer, *n* = 662 in the autumn and *n* = 686 in the winter. Different letters in the same rows are significant (*p* < 0.05).

**Table 2:** Change in the blood melatonin levels in dairy cows (ng/mL).

Day	0 d	1 d	2 d	3 d	4 d	15 d
MT	9.3 ± 1.15 ^d^	17.7 ± 1.31 ^α^^c^	21.6 ± 1.37 ^α^^b^	23.8 ± 2.24 ^α^^b^	37.4 ± 2.68 ^a^	10.3 ± 2.34 ^d^
control	8.4 ± 1.32	6.5 ± 2.22 ^β^	8.8 ± 1.45 ^β^	7.5 ± 1.53 ^β^	9.2 ± 1.43 ^β^	8.9 ± 1.56

All data are represented by the mean ± standard error (*n* = 20). In the same rows, different letters represent a significance (*p* < 0.05). In the same columns, different Greek letters (α and β) represent a significance (*p* < 0.05). MT: melatonin.

**Table 3:** Effects of the treatment with melatonin on the DHI in cows with high somatic cell scores (SCS) during different seasons.

Items	Treatment	Spring	Summer	Autumn	Winter
somatic cell score	Before MT	6.0 ± 0.08 ^α^	6.1 ± 0.12 ^α^	6.0 ± 0.11 ^α^	6.1 ± 0.11 ^α^
	After MT	4.9 ± 0.15 ^aβ^	4.1 ± 0.20 ^cβ^	4.6 ± 0.16 ^bβ^	4.3 ± 0.17 ^bcβ^
milk yield (kg/d)	Before MT	32.1 ± 1.04 ^ab^	34.1 ± 1.06 ^a^	32.9 ± 1.02 ^ab^	31.7 ± 1.11 ^b^
	After MT	32.1 ± 1.15	32.7 ±1.00	32.1 ± 1.05	31.8 ±1.17
Protein (%)	Before MT	3.5 ± 0.04 ^a^	3.5 ± 0.06 ^a^	3.5 ± 0.05 ^aα^	3.39 ± 0.05 ^b^
	After MT	3.5 ± 0.04 ^ab^	3.5 ± 0.04 ^ab^	3.6 ± 0.05 ^aβ^	3.43 ± 0.05 ^b^
Lactose (%)	Before MT	4.9 ± 0.02 ^bα^	5.0 ± 0.03 ^aα^	5.0 ± 0.03 ^aα^	4.7 ± 0.07 ^c^
	After MT	4.9 ± 0.02 ^bβ^	5.1 ± 0.02 ^aβ^	5.1 ± 0.04 ^aβ^	4.8 ± 0.04 ^c^
fat (%)	Before MT	5.2 ± 0.12 ^aα^	4.5 ± 0.16 ^c^	4.8 ± 0.11 ^b^	4.8 ± 0.10 ^b^
	After MT	4.8 ± 0.14 ^aβ^	4.2 ± 0.12 ^b^	4.7 ± 0.13 ^a^	4.8 ± 0.11 ^a^
dry matter (%)	Before MT	14.2 ± 0.14 ^a^	13.6 ± 0.17 ^b^	14.0 ± 0.13 ^a^	13.7 ± 0.10 ^b^
	After MT	13.9 ± 0.15 ^b^	13.4 ± 0.13 ^c^	14.1 ± 0.14 ^a^	13.7 ± 0.12 ^b^
urea nitrogen (mg/dL)	Before MT	13.1 ± 0.39 ^b^	16.6 ± 0.64 ^a^	12.1 ± 0.43 ^b^	17.4 ± 0.47 ^a^
	After MT	14.4 ± 0.45 ^b^	14.2 ± 0.50 ^b^	11.7 ± 0.36 ^c^	15.9 ± 0.51 ^a^

All data are represented by the mean ± standard error, (*n* = 90 in the spring, *n* = 74 in the summer, *n* = 83 in the autumn and *n* = 81 in the winter). In the same rows, different letters represent a significance (*p* < 0.05). In the same columns, different Greek letters (α and β) represent a significance (*p* < 0.05).

**Table 4:** DHI changes of Holstein cows at different ages.

Items	Two	Three	Four	Five	Six	Seven
somatic cell score	4.0 ± 0.24 ^d^	4.3 ± 0.21 ^cd^	4.9 ± 0.15 ^ab^	4.8 ± 0.20 ^abc^	4.7 ± 0.19 ^bc^	5.3 ± 0.17 ^a^
milk yield (kg/d)	32.9 ± 0.58 ^bc^	35. 7 ± 0.66 ^a^	31.9 ± 0.34 ^cd^	34.7 ± 0.56 ^ab^	34.3 ± 0.75 ^ab^	30.2 ± 1.57 ^d^
Protein (%)	3.3 ± 0.03 ^c^	3.4 ± 0.02 ^bc^	3.5 ± 0.50 ^a^	3.5 ± 0.07 ^ab^	3.4 ± 0.05 ^abc^	3.5 ± 0.75 ^abc^
Lactose (%)	5.2 ± 0.02 ^a^	5.1 ± 0.02 ^a^	5.0 ± 0.05 ^bc^	4.8 ± 0.06 ^c^	5.0 ± 0.03 ^b^	4.9 ± 0.04 ^bc^
fat (%)	4.5 ± 0.14 ^b^	4.5 ± 0.10 ^b^	4.7 ± 0.12 ^ab^	4.8 ± 0.08 ^ab^	4.9 ± 0.14 ^a^	4.5 ± 0.24 ^ab^
dry matter (%)	13.6 ± 0.14 ^a^	13.6 ± 0.10 ^a^	14.0 ± 0.15 ^a^	13.9 ± 0.10 ^a^	14.0 ± 0.13 ^a^	13.6 ± 0.21 ^a^
urea nitrogen (mg/dL)	14.4 ± 0.86 ^a^	14.0 ± 0.15 ^a^	14.6 ± 1.01 ^a^	14.7 ± 1.04 ^a^	15.2 ± 1.09 ^a^	14.9 ± 1.37 ^a^

All the data are represented by the mean ± standard error (2 years old: *n* = 507, 3 years old: *n* = 681, 4 years old: *n* = 601, 5 years old: *n* = 502, 6 years old: *n* = 159 and 7 years old: *n* = 62). Different letters in the same rows are significant (*p* < 0.05), and the same letters are not significant (*p* > 0.05).

**Table 5:** Effects of melatonin on the DHI in cows with high SCS during different ages.

Items	Treatment	Two	Three	Four	Five	Six	Seven
Somatic cell score	Before MT	5.3 ± 0.27 ^cα^	5.7 ± 0.15 ^abcα^	5.9 ± 0.17 ^aα^	5.5 ± 0.18 ^bcα^	5.6 ± 0.26 ^abc^	5.9 ± 0.20 ^aα^
	After MT	3.9 ± 0.33 ^dβ^	4.3 ± 0.18 ^cdβ^	4.7 ± 0.18 ^bβ^	4.0 ± 0.19 ^cdβ^	5.2 ± 0.27 ^a^	5.0 ± 0.29 ^abβ^
milk yield (kg/d)	Before MT	32.1 ± 0.95 ^b^	31.7 ± 1.04 ^b^	30.9 ± 1.19 ^b^	35.0 ± 1.54 ^a^	33.7 ± 2.45 ^ab^	33.5 ± 1.73 ^ab^
	After MT	32.1 ± 1.63 ^ab^	32.1 ± 0.96 ^ab^	30.0 ± 1.22 ^b^	33.6 ± 1.52 ^ab^	35.2 ± 2.67 ^a^	30.3 ± 2.08 ^b^
Protein (%)	Before MT	3.4 ± 0.07 ^b^	3.4 ± 0.04 ^b^	3.6 ± 0.06 ^a^	3.4 ± 0.06 ^b^	3.4 ± 0.07 ^b^	3.2 ± 0.06 ^c^
	After MT	3.4 ± 0.09 ^b^	3.4 ± 0.04 ^b^	3.7 ± 0.06 ^a^	3.6 ± 0.07 ^a^	3.4 ± 0.07 ^b^	3.3 ± 0.07 ^b^
Lactose (%)	Before MT	5.2 ± 0.04 ^a^	5.0 ± 0.03 ^bα^	4.8 ± 0.04 ^cα^	4.8 ± 0.04 ^c^	4.9 ± 0.05 ^bc^	4.9 ± 0.06 ^bc^
	After MT	5.2 ± 0.03 ^a^	5.0 ± 0.02 ^bcβ^	4.9 ± 0.03 ^cβ^	4.9 ± 0.05 ^c^	4.9 ± 0.06 ^b^	4.9 ± 0.06 ^b^
Fat (%)	Before MT	4.7 ± 0.24 ^ab^	4.7 ± 0.12 ^bα^	5.0 ± 0.16 ^a^	4.8 ± 0.14 ^ab^	5.1 ± 0.18 ^a^	5.2 ± 0.37 ^a^
	After MT	4.5 ± 0.26 ^bc^	4.4 ± 0.11 ^cβ^	4.7 ± 0.13 ^b^	4.6 ± 0.16 ^bc^	5.3 ± 0.21 ^a^	4.7 ± 0.37 ^abc^
dry matter(%)	Before MT	13.9 ± 0.22 ^ab^	13.8 ± 0.12 ^b^	14.2 ± 0.17 ^a^	13.8 ± 0.17 ^b^	14.1 ± 0.20 ^ab^	13.8 ± 0.36 ^b^
	After MT	13.9 ± 0.27 ^ab^	13.6 ± 0.13 ^b^	14.2 ± 0.15 ^a^	13.9 ± 0.18 ^ab^	14.1 ± 0.24 ^a^	13.5 ± 0.32 ^b^
urea nitrogen (mg/dL)	Before MT	12.8 ± 0.90 ^c^	14.1 ± 0.53 ^b^	14.3 ± 0.61 ^ab^	15.0 ± 0.52 ^ab^	15.4 ± 0.90 ^ab^	16.5 ± 1.64 ^a^
	After MT	13.9 ± 0.80 ^bc^	13.6 ± 0.46 ^bc^	13.3 ± 0.49 ^c^	14.1 ± 0.59 ^bc^	15.8 ± 0.94 ^a^	15.1 ± 1.34 ^ab^

All the data are represented by the mean ± standard error (2 years old: *n* = 23, 3 years old: *n* = 80, 4 years old: *n* = 66, 5 years old: *n* = 56, 6 years old: *n* = 27 and 7 years old: *n* = 19). In the same rows, different letters represent a significance (*p* < 0.05). In the same columns, different Greek letters (α and β) represent a significance (*p* < 0.05).

**Table 6:** The DHI changes of Holstein cows at different lactation stages.

Items	Peak Lactation Stage	Middle Lactation Stage	End Lactation Stage
somatic cell score	2.3 ± 0.31 ^b^	3.1 ± 0.25 ^ab^	3.1 ± 0.19 ^a^
milk yield (kg/d)	33.2 ± 2.09 ^a^	38.9 ± 1.19 ^a^	32.1 ± 0.93 ^b^
Protein (%)	3.4 ± 0.05 ^a^	3.4 ± 0.03 ^a^	3.4 ± 0.03 ^a^
Lactose (%)	5.2 ± 0.04 ^a^	5.2 ± 0.03 ^a^	5.2 ± 0.02 ^a^
fat (%)	4.4 ± 0.15 ^a^	4.1 ± 0.11 ^a^	4.0 ± 0.08 ^a^
dry matter (%)	13.7 ± 0.14 ^a^	13.4 ± 0.12 ^a^	13.3 ± 0.08 ^a^
urea nitrogen (mg/dL)	12.1 ± 0.51 ^a^	12.5 ± 0.50 ^a^	11.8 ± 0.33 ^a^

All data are expressed by the mean ± standard error, (*n* = 21 during peak lactation, *n* = 54 during middle lactation and end lactation: *n* = 98). Different letters in the same rows are significant (*p* < 0.05), and the same letters are not significant (*p* > 0.05).

**Table 7:** Effects of melatonin on the DHI in cows with high SCS during different lactation stages.

Items	Treatment	Peak Lactation	Middle Lactation	End Lactation
somatic cell score	Before MT	2.9 ± 0.32 ^b^	3.9 ± 0.25 ^aα^	4.3 ± 0.18 ^aα^
	After MT	2.0 ± 0.24	2.0 ± 0.17^β^	2.0 ± 0.15^β^
milk yield (kg/d)	Before MT	32.9 ± 2.34 ^b^	40.0 ± 1.35 ^a^	31.6 ± 1.22 ^b^
	After MT	38.8 ± 2.06 ^a^	39.1 ± 1.79 ^a^	31.3 ± 1.29 ^b^
Protein (%)	Before MT	3.5 ± 0.06	3.5 ± 0.04	3.5 ± 0.04
	After MT	3.4 ± 0.06 ^ab^	3.5 ± 0.05 ^a^	3.4 ± 0.04 ^b^
Lactose (%)	Before MT	5.2 ± 0.05 ^aα^	5.1 ± 0.03 ^b^	5.1 ± 0.03 ^bα^
	After MT	5.3 ± 0.04 ^aβ^	5.2 ± 0.02 ^b^	5.2 ± 0.02 ^bβ^
Fat (%)	Before MT	4.5 ± 0.19 ^a^	4.2 ± 0.12 ^b^	4.1 ± 0.08 ^b^
	After MT	3.9 ± 0.26	4.2 ± 0.11	4.2 ± 0.12
dry matter (%)	Before MT	13.8 ± 0.16 ^a^	13.5 ± 0.14 ^ab^	13.4 ± 0.09 ^b^
	After MT	13.2 ± 0.29 ^b^	13.6 ± 0.14 ^a^	13.5 ± 0.13 ^a^
urea nitrogen (mg/dL)	Before MT	11.9 ± 0.65 ^a^	12.7 ± 0.65 ^a^	11.5 ± 0.38 ^a^
	After MT	11.1 ± 0.64 ^a^	12.1 ± 0.54 ^a^	12.0 ± 0.38 ^a^

All data are expressed by the mean ± standard error (*n* = 15 at the peak, *n* = 38 at the middle and *n* = 59 at the end of lactation). In the same rows, different letters represent a significance (*p* < 0.05). In the same columns, different Greek letters (α and β) represent a significance (*p* < 0.05).

**Table 8:** The number of the healthy cows on different cattle farms.

Farm 1	Lactation Period	Peak	Middle	Late			
(summer, age 4)	Number	21	54	98			
Farm 2	Seasons	Spring	Summer	Autumn	Winter		
(middle, ages 3 to 4)	Number	728	702	662	686		
Farm 3	Age	Two	Three	Four	Five	Six	Seven
(summer, middle)	Number	507	681	601	502	159	62

**Table 9:** The number of cows that received the melatonin treatment on different cattle farms.

Farm 1	Lactation Period	Peak	Middle	Late			
(summer, age 4)	Number	15	38	59			
Farm 2	Seasons	Spring	Summer	Autumn	Winter		
(middle, ages 3 to 4)	Number	90	74	83	81		
Farm 3	Age	Two	Three	Four	Five	Six	Seven
(summer, middle)	Number	23	80	66	56	27	19

Note: Peak lactation period: 16–100 days, middle lactation period: 101–200 days and end of lactation: 201 days—no milk.

## Data Availability

The data presented in this study are available on request from the corresponding author.

## Sample Availability

Samples of the compounds are not available from the authors.
